# Mid-long-term follow-up of operated level kinematics after single-level artificial cervical disc replacement with Bryan disc

**DOI:** 10.1186/s13018-022-03051-2

**Published:** 2022-03-09

**Authors:** Chuanhong Li, Xing Yu, Yang Xiong, Yongdong Yang, Fengxian Wang, He Zhao

**Affiliations:** 1 Department of Orthopedics, Dongzhimen Hospital, Beijing University of Chinese Medicine, No. 5 Haiyuncang Street, Dongcheng District, Beijing, 100700 China; 2 School of Materials Science and Engineering, Tsinghua University, Beijing, 100084 China

**Keywords:** Artificial cervical disc replacement, Bryan disc, Center of rotation, Kinematics, Clinical outcomes, Radiological outcomes

## Abstract

**Objective:**

Evaluation of the mid-long-term kinematics of single-level Bryan artificial cervical disc replacement (ACDR) in vivo by analyzing the center of rotation (COR) at the operated level.

**Methods:**

A retrospective analysis was conducted using data collected from 38 patients who underwent single-level Bryan ACDR from January 2010 to March 2013. Radiological parameters including range of motion (ROM), lordosis angle, translation, and COR were obtained. Clinical outcomes were assessed based on Odom Criteria, modified Japanese Orthopedic Association (mJOA), Neck Disability Index (NDI), and Visual Analogue Scale (VAS) scores. Correlations between COR and other follow-up data were discussed at the last follow-up.

**Results:**

Compared with preoperative values, the last follow-up data showed that 86.84% of cases achieved good-or-excellent outcomes based on Odom criteria; Significant improvements were observed across all scales assessed for clinical outcomes (*P* < 0.05); Lordosis angle was significantly increased in both the overall cervical spine and the operated level (*P* < 0.05); ROM of the overall cervical spine, operated level, and adjacent levels was preserved (*P* > 0.05); There was no significant change in COR at the operated level (*P* > 0.05). At the last follow-up and at the operated level, COR (*Y*) showed negative correlations with ROM and translation (*P* < 0.05), but no follow-up data correlated with COR (*X*) were found (*P* > 0.05).

**Conclusions:**

Satisfactory clinical and radiological outcomes were achieved 7 years or more after single-level Bryan ACDR. At the operated level, preoperative COR was maintained, probably due to replicating the physiological interrelations of COR (*Y*), translation, and ROM.

## Introduction

Artificial cervical disc replacement (ACDR) is a procedure aimed at anterior intervertebral decompression and the subsequent reconstruction of mobility for symptomatic cervical degenerative disc disease (CDDD). Based on data from short- and mid- to long-term follow-up studies, ACDR has shown similar clinical outcomes to anterior cervical discectomy and fusion (ACDF). Moreover, since motion at the operated level can be preserved, it is considered that ACDR may delay or even prevent adjacent segment degeneration (ASD) through reduction of mechanical load and compensatory motion at the adjacent levels compared with cervical fusion [1, 2]. A number of publications have already demonstrated the successful preservation of range of motion (ROM) at the operated level, but the purpose of ACDR is to restore the cervical physiological motion after nerve decompression, and the physiological motion patterns may influence the long-term clinical outcomes of ACDR through prolonged viability of the implants, reduction of stress at zygapophyseal joints, and improvement of the kinematics and biomechanics at adjacent levels [3–5]. Thus, attention is increasingly shifting to in vivo kinematics analysis of the artificial cervical disc [6].

Due to the lack of a simple and effective method to evaluate three-dimensional cervical motion, cervical kinematics studies are mainly based on sagittal flexion–extension motion [7]. The center of rotation (COR) is the sagittal motion center of the functional spinal unit (FSU) measured using cervical flexion–extension radiographs [7, 8]. COR is relatively simple to measure and can be readily subjected to statistical analysis and comparison. Unlike ROM, which amounts to the sum of declination angles generated by intervertebral rotation and translation, COR constitutes a combination of sagittal motion informations of the cervical FSU and enables the delineation of the quality and trajectory of intervertebral motion [9, 10], interpretation of the biomechanical environment and stability of the index level [4, 11, 12], and detection of irregular motion patterns of the FSU when ROM does not show any abnormality [13]. Due to these advantages, COR has been commonly adopted for kinematics analysis after ACDR [14, 15].

The Bryan cervical disc (Medtronic Sofamor Danek, Memphis, TN, USA), with its ability to simulate physiological kinematics, was once one of the most widely used artificial cervical discs in cervical spine surgery. Satisfactory clinical outcomes have been shown in 10- [16], 15- [17], and 18-year [18] follow-up studies for Bryan ACDR. However, detailed postoperative kinematics analysis of mid- to long-term follow-up is needed, and the factors that may affect the location of COR at the operated level remain to be determined. This study is aimed at a mid-long-term (at least 7 years) kinematics analysis of single-level Bryan ACDR and the investigation of potential factors correlated to COR with data gathered at the last follow-up.

## Materials and methods

### Patient populations

This study is a retrospective analysis of consecutive cases that underwent single-level Bryan ACDR in our hospital between January 2010 and March 2013. Inclusion criteria are: (1) patients were diagnosed with symptomatic CDDD based on radiological and clinical findings and failed to show improvement after non-surgical treatment for over 6 weeks; (2) age of patients was between 30 and 60 years; (3) operations were performed by the same surgeon; (4) the follow-up period was longer than 84 months. Exclusion criteria are: (1) patients with the following conditions were not considered as suitable candidates for ACDR: evident cervical instability, severe collapse of intervertebral space, marked reduction of ROM, cervical spinal bony stenosis, etc.; (2) patients received secondary operation. This study was approved by the Medical Ethics Committee of Dongzhimen Hospital (ethical approval number: 2021DZMEC-082-02). All patients were informed in advance and voluntarily consented to participate in the study.

### Assessment of clinical outcomes

The modified Japanese Orthopedic Association (mJOA), visual analogue scale (VAS), and Neck Disability Index (NDI) scores were determined preoperatively and postoperatively at least 7 years in order to assess the clinical outcomes of Bryan ACDR. The mJOA score was used to assess motor, sensory, and sphincter dysfunction. Neck and arm pain related to CDDD were quantified using VAS score, and limitations in daily activities were assessed with NDI score. The Odom Criteria was used at the last follow-up to rate the clinical outcomes as follows: (a) excellent, all preoperative symptoms relieved, daily life and occupation not impaired; (b) good, occasional reemergence or minimal persistence of preoperative symptoms but no significant interference with daily occupational tasks; (c) satisfactory, relief of some preoperative symptoms, but daily occupational tasks and activities remain significantly impaired; (d) poor, symptoms and signs unimproved or exacerbated. Two formulas were used to calculate the improvement rate of clinical outcomes: the mJOA improvement rate, [(postoperative score − preoperative score)/(17 − preoperative score)] * 100%; the NDI or VAS improvement rate, [(preoperative score − postoperative score)/preoperative score] * 100%.

### Radiographic assessment

The cervical radiographs in neutral–lateral and flexion–extension positions were collected before and 7 years or more after operation. Radiological parameters were measured independently by two orthopedic surgeons using Mimics 17.0 (Materialise, Leuven, Belgium) and ImageJ (Wayne Rasband, National Institutes of Health, USA) software. Every parameter was measured 3 times, and the average was used for further analysis.

**Fig. 1:**
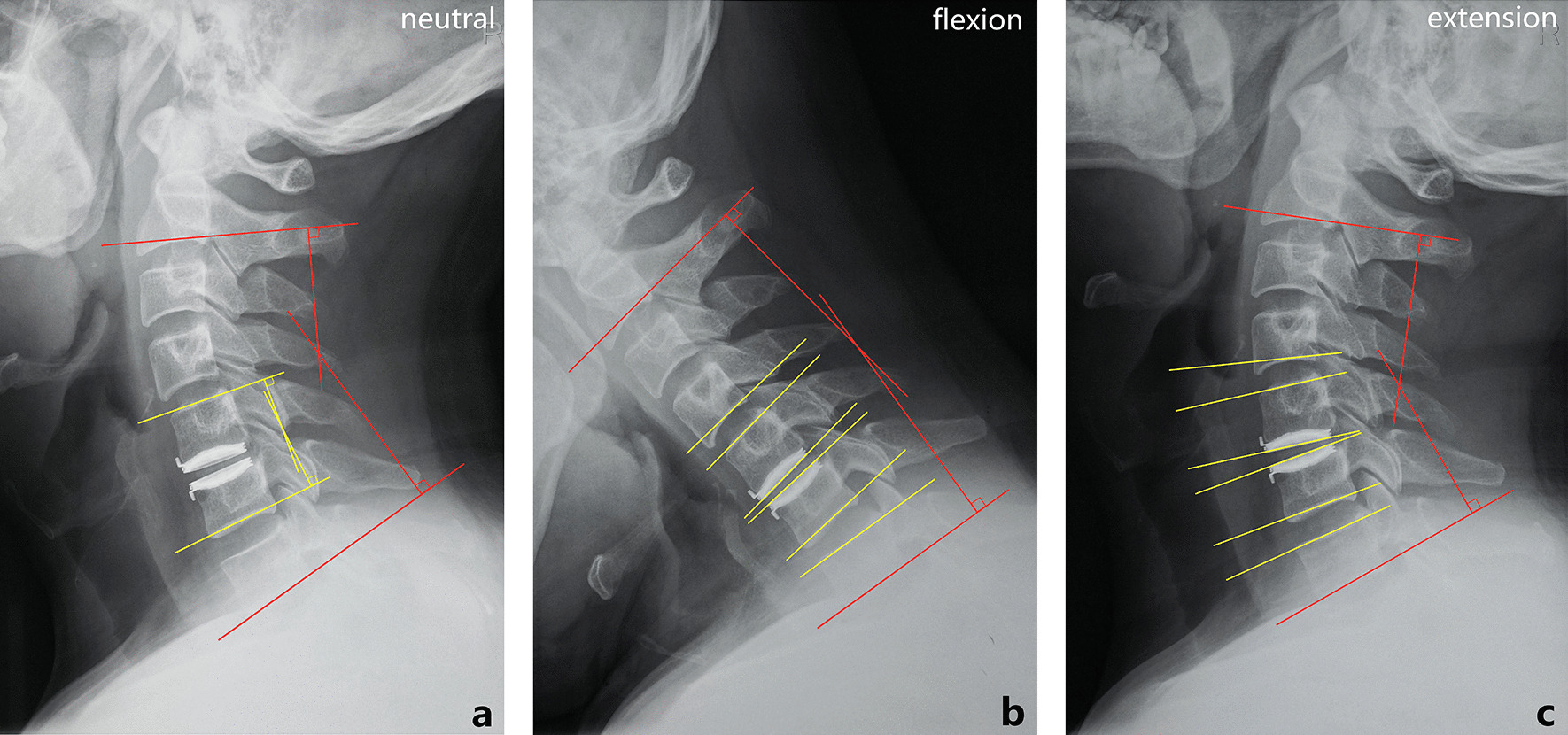
Radiographic measurements using the Cobb’s angle. **a** In the neutral–lateral radiograph, the lordosis angle of the overall cervical spine (red lines) and operated level (yellow lines) was defined as the angles between the tangents of the corresponding vertebral endplates. A positive value was assigned to an angle with an anterior opening, while the negative value presented the posterior opening. **b**, **c** In the flexion–extension radiographs, the overall cervical ROM and segmental ROM (ROM of the operated level, SROM, and IROM) were the flexion–extension difference of the overall cervical lordosis angle and intervertebral angle (between the two yellow lines), respectively

**Fig. 2:**
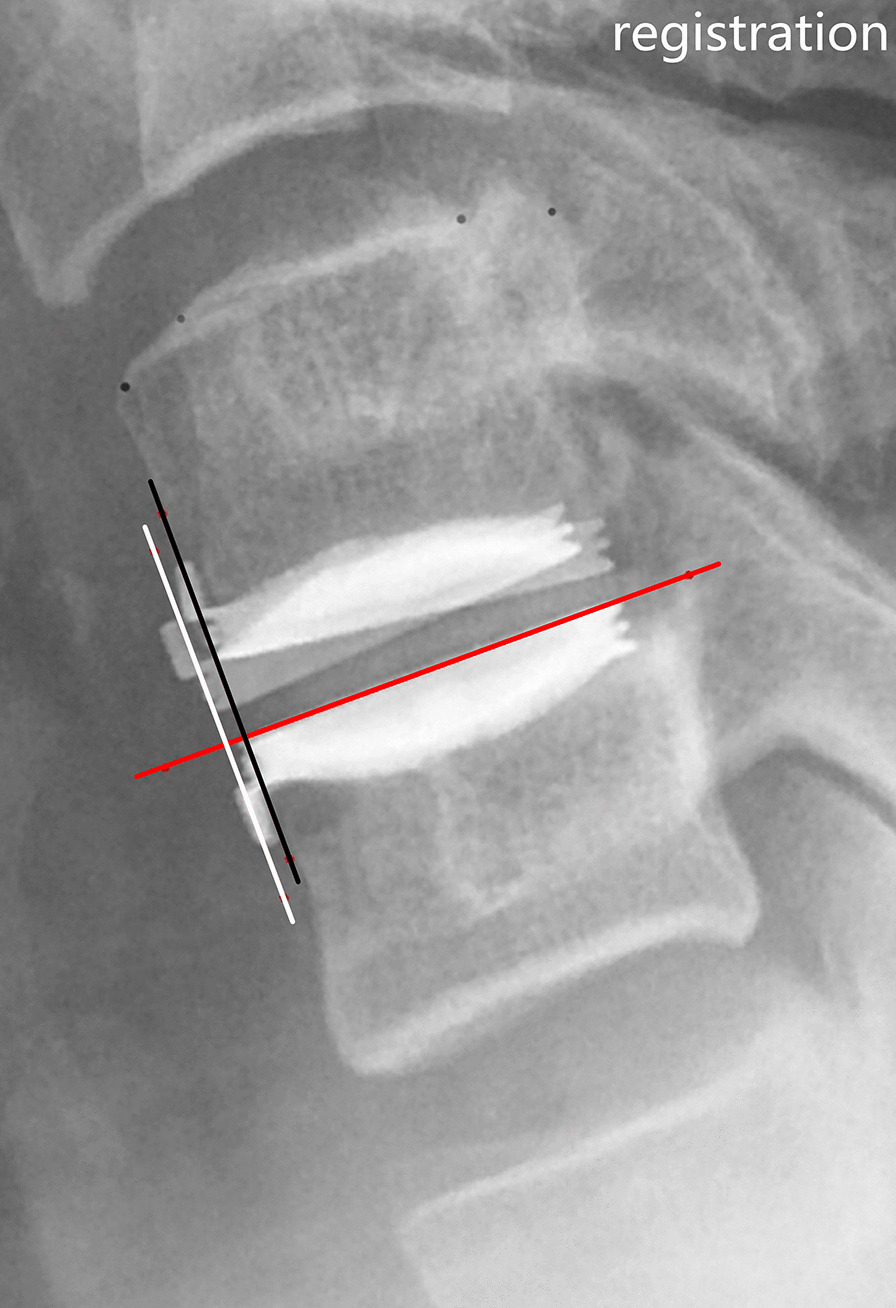
Measurement of the translation at the operated level. The flexion and extension radiographs were superimposed by aligning the inferior vertebrae. The tangent (in red) of the inferior endplate of the prosthesis was made, and the two lines perpendicular to the red line, touching the inferior anterior tip of the superior endplate of the prosthesis in flexion (line in white) and extension (line in black), were erected. The vertical distance between these two parallel lines was determined as the translation

The Cobb’s angle was applied to determine the lordosis angle of the overall cervical spine (C2–C7) and operated level in a neutral–lateral radiograph, as well as the ROM of the overall cervical spine (C2–C7), operated level, and adjacent levels (superior range of motion, SROM; inferior range of motion, IROM) in flexion–extension radiographs (Fig. 1). The translation at the operated level was quantified by measuring the sagittal displacement distance of the inferior anterior tip of the prosthesis’s superior endplate along the parallel line of the prosthesis’s inferior endplate during flexion and extension (Fig. 2) [19–21].

**Fig. 3:**
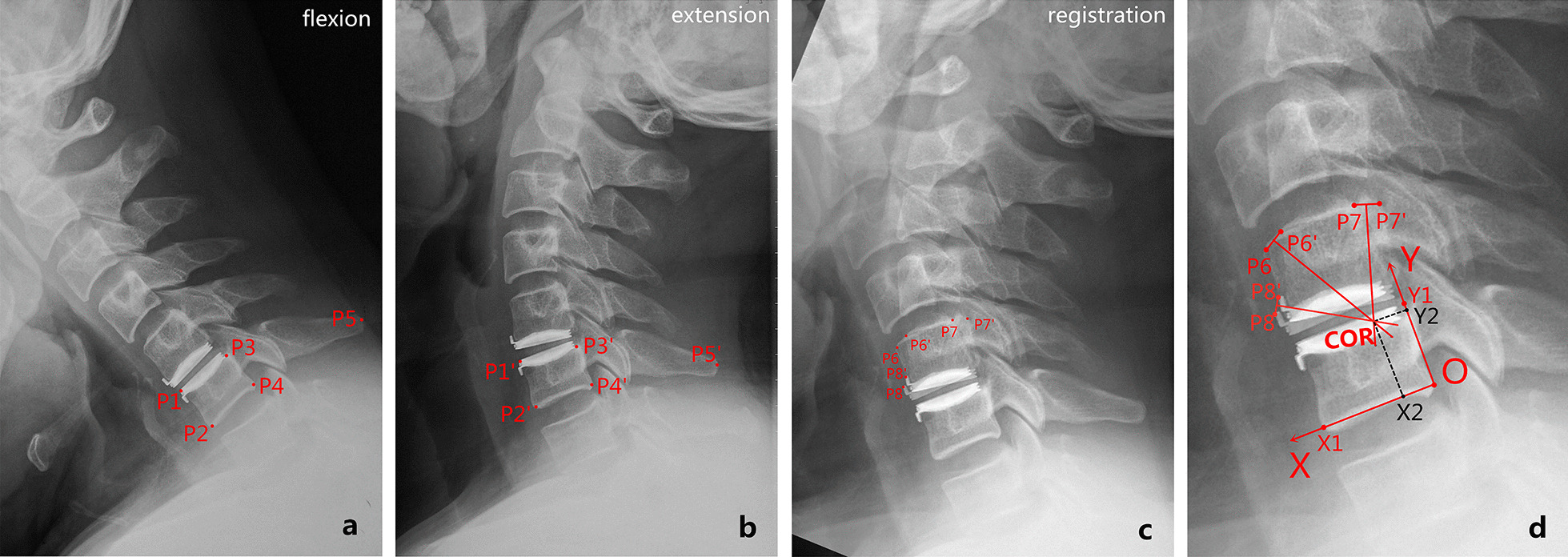
Measurement of the COR at the operated level (C5–C6). **a**–**c** Aided by the alignments between P1–P5 and P1'–P5' in C6 vertebra, the flexion and extension radiographs were superimposed. **c** Three anatomical landmarks in C5 vertebra were located and labeled as P6–P8 in the flexion. Similarly, the corresponding markers were labeled as P6'–P8' in the extension. **d** COR was determined as the converging point of the three perpendicular bisectors (corresponding to segments P6P6', P7P7', and P8P8'). Referring to the procedures outlined by Amevo et al. [26], a rectangular coordinate system was set up using the tangents of C6 vertebral body. The forward and upward directions are positive directions of the *X*- and *Y*-axis, respectively. OX1 and OY1 correspond to the width and height of C6 vertebral body, respectively. The location of COR is noted as the following, COR (*X*, *Y*) = [(OX2/OX1) * 100%, (OY2/OY1) * 100%]

The COR at the operated level was determined using the method of perpendicular bisectors based on Euler’s rotation theorem [22]. The sagittal motion trajectory of the superior vertebra relative to the inferior vertebra within an FSU is considered as an arc surrounding COR, and the COR can be determined through geometric measurement by: (1) superimposing the flexion and extension radiographs with the alignment of the inferior vertebrae; (2) connecting corresponding anatomical landmarks in the two superior vertebrae with a straight line and erecting perpendicular bisectors for those lines; (3) determining the point where all perpendicular bisectors meet. This point corresponds to the COR (Fig. 3) [23, 24]. To minimize technical errors, the following procedures were implemented: (1) cases with ROM < 5° at the operated level were not included due to the difficulties involved in locating and connecting the set of markers in the radiographs [25]; (2) the automatic registration function in Mimics 17.0 software was used to accurately superimpose the inferior vertebrae of the target FSU in the flexion–extension radiographs [24]; (3) a rectangular coordinate system was established around the inferior vertebral body in order to describe the location of COR, and the *X*- and *Y*-axis were aligned to distinct profiles of the vertebral body following the methods described by Amevo et al. [26]. The horizontal and vertical coordinates of COR were normalized against the width and height of the inferior vertebral body [27].

### Statistical analysis

The SPSS 20.0 software (SPSS, Chicago, IL, USA) was used for statistical analysis of all the data. The results were presented as mean ± standard deviation (SD). Paired *t*-test and Pearson’s correlation analysis were applied to test for significant differences and correlations, respectively. *P* < 0.05 was considered statistically significant.

## Results

Follow-up data of 41 patients were obtained. At the last follow-up, 3 patients developed grade III–IV heterotopic ossification and ROM < 5° at the operated level. These 3 cases were excluded due to large errors in measuring COR [25], and a total of 38 patients (18 males and 20 females) were eventually included in the study, with a mean age of 46.86 ± 6.91 years (range 34–59 years) at index surgery. Preoperative symptoms were attributed to myelopathy (24 cases), radiculopathy (7 cases), and myeloradiculopathy (7 cases). Operated levels included C3–C4 (1 case), C4–C5 (15 cases), C5–C6 (21 cases), and C6–C7 (1 case). The mean follow-up was 93.97 ± 9.67 months (range 84–118 months). Basic information about the patients is listed in Table 1.

**Table 1:** Basic information

Variable	Value
Number of patients, *n*	38
*Sex, n (%)*	
Male	18 (47.37%)
Female	20 (52.63%)
Age, years, mean ± SD (range)	46.86 ± 6.91 (34–59)
*Symptoms, n (%)*	
myelopathy	24 (63.16%)
radiculopathy	7 (18.42%)
myeloradiculopathy	7 (18.42%)
*Index level, n (%)*	
C3–C4	1 (2.63%)
C4–C5	15 (39.47%)
C5–C6	21 (55.27%)
C6–C7	1 (2.63%)
Follow-up, months, mean ± SD (range)	93.97 ± 9.67 (84–118)

### Clinical outcomes

At the last follow-up, sensory, motor, and sphincter dysfunction caused by CDDD were significantly improved, and neck and arm pain were significantly alleviated. In contrast to preoperative values, the mJOA score was significantly increased (*P* < 0.05) with an 80.62 ± 13.59% rate of improvement; the NDI and VAS (neck and arm pain) scores were significantly reduced (*P* < 0.05) with a mean improvement rate of 72.18 ± 12.81%, 76.73 ± 16.95%, and 76.15 ± 9.06%, respectively (Table 2). 86.84% of patients ranked in the “excellent” or “good” category based on the Odom criteria at the last follow-up (Excellent, 23 cases; Good, 10 cases; Satisfactory, 5 cases; Poor, 0 cases).

**Table 2:** A summary of clinical outcomes

Parameter	Preoperation	Last follow-up	*P* value
mJOA	10.08 ± 3.05	15.47 ± 1.20*	< 0.001
VAS (neck pain)	5.42 ± 2.10	1.53 ± 0.89*	< 0.001
VAS (arm pain)	5.18 ± 2.52	1.26 ± 0.92*	< 0.001
NDI	30.37 ± 8.24	7.53 ± 3.94*	< 0.001

MJOA, modified Japanese Orthopedic Association; VAS, visual analogue scale; NDI, Neck Disability Index

^*^P < 0.05 compared with preoperation

### Radiological outcomes

As shown in Table 3, overall cervical ROM, ROM of the operated level, SROM, and IROM were well preserved and showed no significant difference compared with preoperative values (*P* > 0.05). Cervical lordosis was partially restored, and the lordosis angle of the overall cervical spine and the operated level increased from 11.29 ± 7.51° and 3.18 ± 4.60° preoperatively to 18.44 ± 9.62° and 5.64 ± 4.50° at the last follow-up, respectively (*P* < 0.05).

**Table 3:** A summary of radiological parameters

Parameters	Preoperation	Last follow-up	*P* value
ROM (overall cervical) (°)	44.26 ± 12.95	46.68 ± 11.93	0.398
Lordosis angle (overall cervical) (°)	11.29 ± 7.51	18.44 ± 9.62*	0.001
Lordosis angle (operated level) (°)	3.18 ± 4.60	5.64 ± 4.50*	0.036
ROM (operated level) (°)	8.73 ± 4.12	10.24 ± 3.45	0.161
SROM (°)	11.88 ± 4.69	11.83 ± 4.02	0.944
IROM (°)	7.22 ± 3.75	8.29 ± 4.85	0.426
COR (*X*) (%)	39.76 ± 17.94	43.24 ± 16.55	0.639
COR (*Y*) (%)	70.16 ± 16.33	77.19 ± 22.54	0.070

ROM, range of motion; SROM, superior range of motion; IROM, inferior range of motion; COR, center of rotation

^*^P < 0.05 compared with preoperation

The location of COR at the operated level was maintained, and COR (*X*) and COR (*Y*) showed no significant difference between preoperation and the last follow-up (*P* > 0.05) (Table 3). The COR (*Y*) at the operated level showed negative correlations with the ROM and translation at the same level (*r* =  − 0.622, *P* < 0.05; *r* =  − 0.767, *P* < 0.05), but no correlation with clinical outcomes and remaining radiological parameters (*P* > 0.05). No correlation was found between the COR (*X*) and any of the parameters analyzed in this study (*P* > 0.05) (Table 4). Moreover, the ROM of the operated level was positively correlated to translation at the same level (*r* = 0.772, *P* < 0.05).

**Table 4:** Correlations between COR and other follow-up data at the last follow-up

Parameters	COR (*X*) (%)		COR (*Y*) (%)	
	*r* value	*P* value	*r* value	*P* value
Age (years)	0.244	0.201	− 0.276	0.147
Follow-up period (months)	0.003	0.989	0.038	0.845
mJOA	− 0.266	0.163	0.213	0.268
VAS (neck pain)	0.036	0.853	0.025	0.897
VAS (arm pain)	0.167	0.388	0.042	0.831
NDI	0.277	0.146	− 0.083	0.668
mJOA improvement rate (%)	− 0.279	0.143	0.184	0.340
VAS (neck pain) improvement rate (%)	0.001	0.998	− 0.068	0.726
VAS (arm pain) improvement rate (%)	− 0.143	0.461	− 0.032	0.868
NDI improvement rate (%)	− 0.354	0.060	− 0.075	0.699
ROM (overall cervical) (°)	− 0.003	0.987	− 0.182	0.346
Lordosis angle (overall cervical) (°)	− 0.108	0.577	− 0.002	0.991
Lordosis angle (operated level) (°)	0.162	0.402	0.132	0.495
ROM (operated level) (°)	− 0.014	0.942	− 0.622*	< 0.001
SROM (°)	0.150	0.438	− 0.078	0.688
IROM (°)	− 0.046	0.811	− 0.269	0.157
Translation (operated level) (mm)	0.263	0.169	− 0.767*	< 0.001

COR, center of rotation; mJOA, modified Japanese Orthopedic Association; VAS, visual analogue scale; NDI, Neck Disability Index; ROM, range of motion; SROM, superior range of motion; IROM, inferior range of motion

^*^P < 0.05, significant correlation between two parameters

## Discussion

The implantation of an artificial cervical disc is aimed at avoiding the interference with cervical biomechanics and kinematics due to the fusion at the operated level and preventing stress concentration and abnormal motion at the adjacent levels. However, it has been sporadically noticed that implantation of the Bryan disc failed to maintain lordosis at the operated level and even caused focal kyphosis [28]. This problem may be the result of suboptimal insertion depth and angle of the prosthesis, uneven- or over-milling of the endplates, or a mismatch in the shape and size of the interface between the prosthesis and the vertebra, and technical improvements targeting these issues are expected to prevent kyphosis at the operated level [20, 29]. In agreement with previous reports [30], this study showed that single-level Bryan ACDR resulted in partial restoration of the physiological lordosis, not only at the operated level but also for the overall cervical spine, while promoting sagittal balance of cervical vertebral alignment. We suggest that the following factors will contribute to the proper restoration of the physiological cervical angle: (1) preoperative screen of indication to exclude patients with segmental instability and kyphosis; (2) preservation of posterior longitudinal ligament and minimization of soft tissue damage during the operation; (3) the milling is parallel to the intervertebral space to avoid overmilling of the anterior and posterior edges of the endplates; (4) postoperative functional training to promote recovery of soft tissue.

In this study, we observed no significant difference of the COR at the operated level 7 years or more after operation compared with that before operation. This observation indicates that the implantation of the Bryan disc did not change the original sagittal motion patterns and therefore ensured the long-term stability of the motion quality at the operated level, which may be the underlying explanation for the observed successful mid-long-term maintenance of the normal cervical biomechanical environment and the favorable clinical outcomes. The ability of the Bryan disc to maintain the COR at the operated level has been confirmed by several clinical reports [3, 15, 31, 32] and finite element analyses [14, 33].

The maintenance of the COR at the operated level is closely associated with the kinematic characteristics of the artificial cervical disc, which are determined by the design and structure of the prosthesis [6]. The Bryan disc is the first truly non-constrained artificial cervical disc used in clinical applications [34]. It contains a proprietary-enclosed nucleus pulposus, which is relatively flexible in sheath and can provide free adjustment of instantaneous center of rotation (ICR) at the operated level during cervical sagittal motion, thus simulating the constant shift of ICR under physiological conditions. This is considered a major factor contributing to the maintenance of the preoperative COR. In contrast, a constrained prosthesis such as the ProDisc-C disc allows only a singular sagittal motion pattern without translation and has a fixed COR/ICR based on the structure of the device, thus resulting in an anterior shift of COR at the operated level if the insertion depth was insufficient [35–37]. The intermediate semi-constrained prosthesis permits small translation and limited sagittal adjustment of ICR. To some degree, these characteristics are able to relax the requirement for the precise location of implantation, but maintenance of preoperative COR is still very difficult to achieve, for example, with the Prestige LP disc [8, 38–40].

**Fig. 4:**
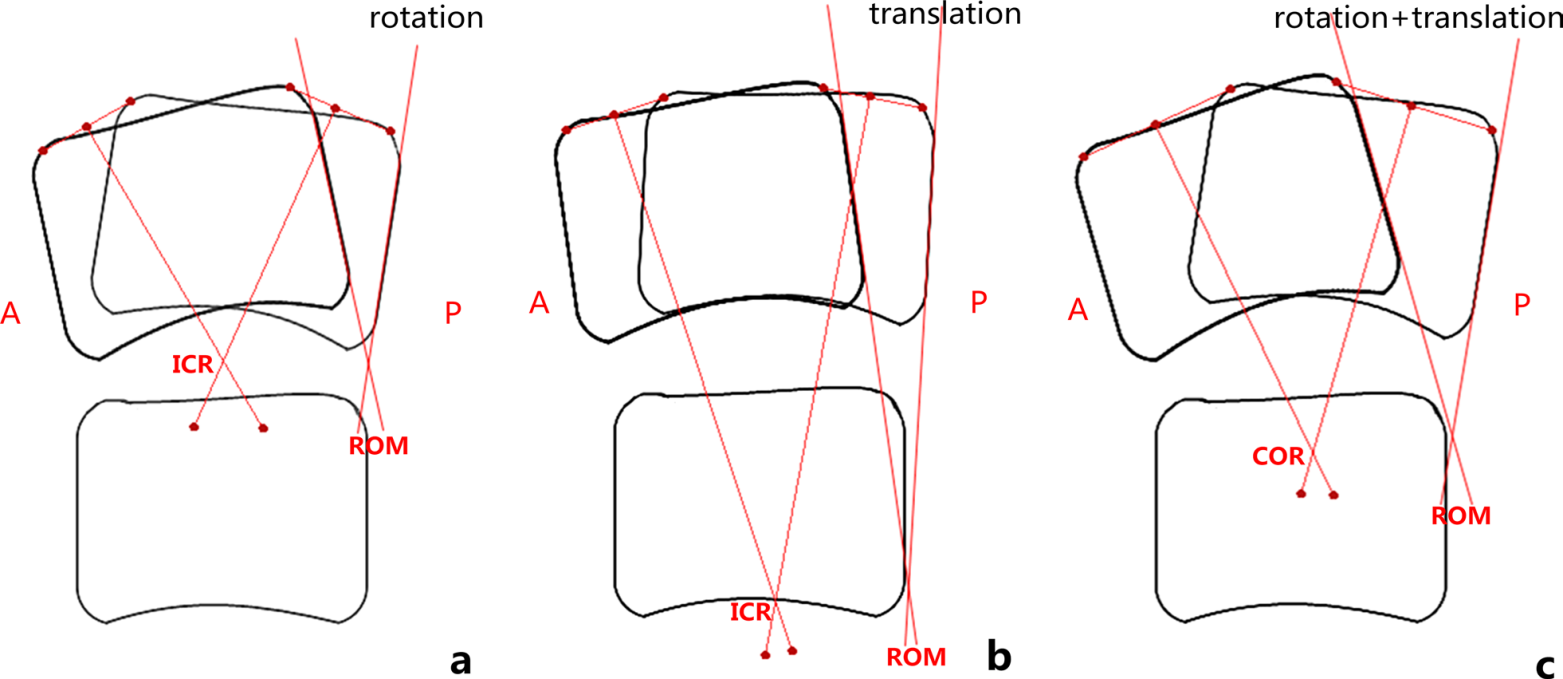
Effects of intervertebral rotation and translation on COR (*Y*). **a**–**c** The angle between the two tangents of the posterior edge of the superior vertebral bodies in flexion and extension positions is defined as the ROM of the target FSU (White method [43]). **a** The ICR is located within the disc in the case of intervertebral motion with only rotation. **b** The ICR is located far below the disc in intervertebral motion with only translation. **c** Because the physiological intervertebral motion consists of both rotation and translation, the COR in the superior–inferior direction is located between the rotation ICR and the translation ICR. A, anterior; P, posterior

**Fig. 5:**
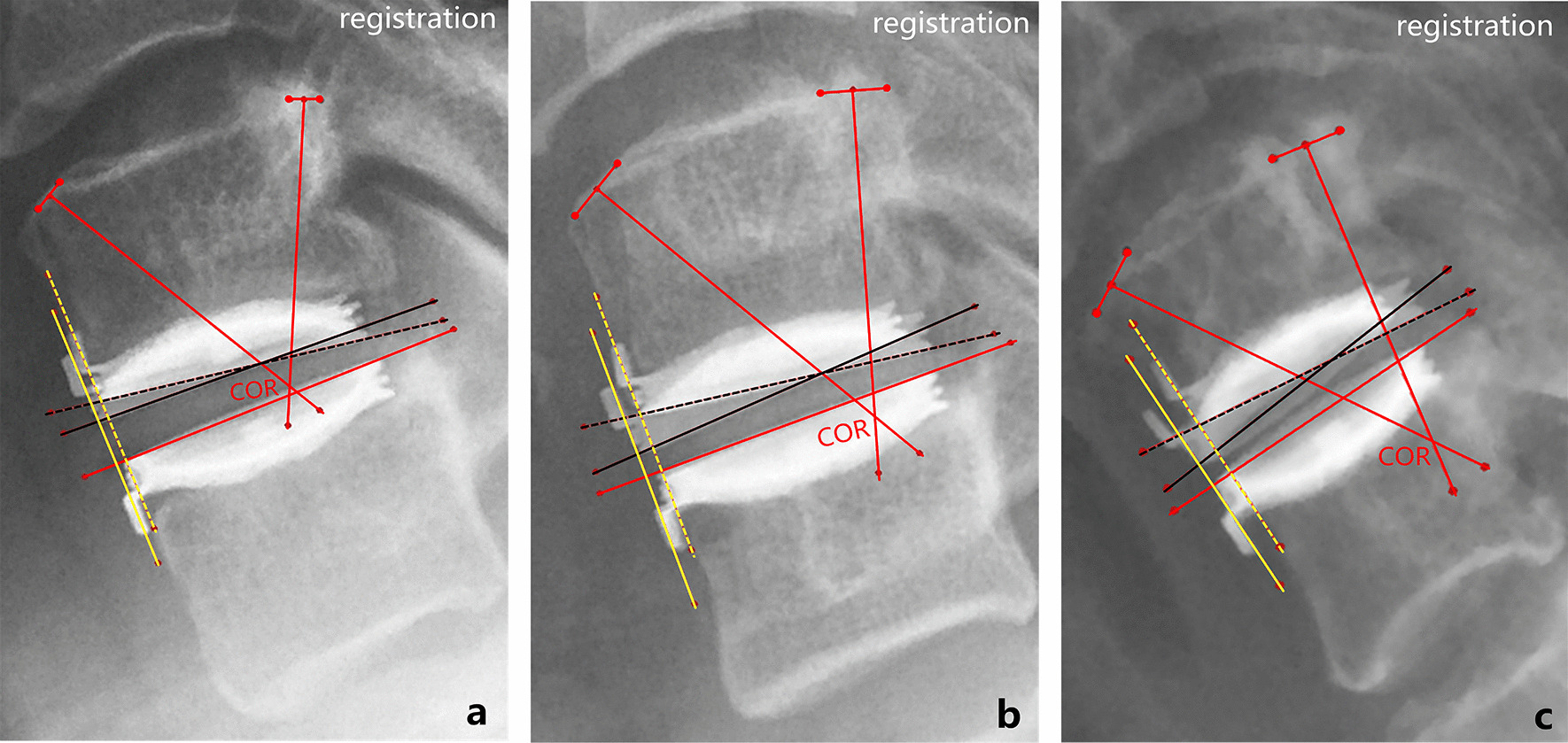
Three representative cases illustrate the interrelations among translation, ROM, and COR (*Y*) at the operated level. **a**–**c** Superimpose the flexion and extension radiographs with the alignment of the inferior vertebrae. The vertical distance between the yellow solid and broken lines represents the translation. The angle between the black solid and broken lines defined as the ROM. **a** Female of 49-year-old patient with C5–C6 as the operated level and 88 months of follow-up. Translation: 0.4 mm; ROM: 6.21°; COR (*Y*): 102.86%. **b** Male of 36-year-old patient with C5–C6 as the operated level and 89 months of follow-up. Translation: 1.1 mm; ROM: 10.05°; COR (*Y*): 90.63%. **c** Female of 51-year-old patient with C5–C6 as the operated level and 93 months of follow-up. Translation: 1.6 mm; ROM: 12.34°; COR (*Y*): 62.23%

At the last follow-up, statistical correlation analysis showed that COR (*Y*) was significantly negatively correlated with ROM and translation at the operated level. A similar correlation between COR (*Y*) and translation from a follow-up study of single-level Discover ACDR was reported by Koller et al. [21]. The cervical intervertebral joint is a 3-joint complex, and the motions of the disc and zygapophyseal joints are coupled. Because the zygapophyseal joint space relative to the intervertebral disc space is tilted at various angles, the sagittal intervertebral motion involves different directional motion components of both translation and rotation [19, 41]. Rotation refers to the rotational motion of the superior vertebra relative to the inferior vertebra around a specific point within the disc (Fig. 4a) [42]. We term this point “rotation ICR”. In contrast, translation is defined as the sliding of the superior vertebra along the superior endplate of the inferior vertebra [43]. However, due to the support of the biconvex disc, the trajectory of translation naturally presents an arc shape, which can be considered as the result of a complex motion of superior–inferior and anterior–posterior translation [19]. Therefore, the translation is actually a rotation around a point far below the disc (Fig. 4b) [42]. This point is termed “translation ICR”. In terms of localization along the superior–inferior direction, the COR is located between the superior rotation ICR and the inferior translation ICR (Fig. 4c), and the COR (*Y*) will change if the proportion of rotation or translation component in the sagittal intervertebral motion is altered. The increase of rotational proportion leads to the superior shift of COR, while the increase in translational proportion leads to the inferior shift of COR [44]. Bogduk et al. [22] summarized the interrelations among translation (*T*), rotation (*θ*), COR (*Y*), and center of reaction (CR) with the following formula: COR (*Y*) = CR (*Y*) − *T*/[2 tan(*θ*/2)]. These are consistent with our results. In other words, for the operated level with rotation as the main component of sagittal intervertebral motion, the COR was located close to the superior endplate of the inferior vertebra and within the disc (Fig. 5a), whereas the COR shifted inferiorly when the translational proportion increased (Fig. 5bc).

The translation causes the superior vertebra to tilt relative to the inferior vertebra in the sagittal plane (Fig. 4b). Therefore, the ROM of the cervical FSU should be defined as the sum of the relative declination angles between two adjacent vertebrae generated by both rotation and translation (Fig. 4c) [43]. Correspondingly, we observed that translation was positively correlated with ROM in this study, and similar findings were described in another follow-up study [21]. Combined with the foregoing findings, it can be seen that with the increase of translation, ROM increases and COR shifts inferiorly. Therefore, at the operated level, we found that greater ROM was associated with lower COR, i.e. there was a negative correlation between COR (*Y*) and ROM. This is clearly demonstrated by the representative cases shown in Fig. 5.

Under the lubrication of normal saline within the sheath, the superior endplate of the Bryan disc slides along the surface of the biconvex nucleus pulposus with an arc motion trajectory during flexion and extension [33], which simulates the physiological translation. After the implantation of the Bryan disc, the balanced and stable interactions between the surrounding soft tissues and the vertebrae or prosthesis can be restored with the overall biomechanical and kinematic adjustments of the cervical spine. Meanwhile, according to physiological needs, with the cooperation of surrounding soft tissues, the arc sliding (translation) of the superior endplate relative to the inferior endplate of the prosthesis can be adjusted automatically during flexion and extension. Therefore, an appropriate ROM of the operated level was obtained, and the COR was adjusted to the optimal location along the superior–inferior direction, so as to simulate the preoperative ROM and COR.

There are some limitations in this study. First, the location of COR at the level with cervical disc degeneration will change, and the preoperative COR may not represent the physiological COR [45]. Second, our analysis was limited to data collected at two time points: preoperation and the last follow-up. A complete trend of clinical and radiological outcomes over time is not available but should be studied. Third, no correlative factors were identified for COR (*X*). However, the formula proposed by Bogduk et al. [22] suggests a positive correlation between COR (*X*) and translation, and the insertion depth of the prosthesis may also affect COR (*X*) [36]. Larger sample sizes and improved clinical research methods are required to advance the kinematic research of ACDR in the future.

## Conclusions

Satisfactory clinical and radiological outcomes for single-level Bryan ACDR were confirmed in this mid-long-term follow-up study. At least 7 years after operation, the COR at the operated level was maintained, probably due to the replication of the physiological interrelations, i.e. COR (*Y*) is negatively correlated to translation and ROM.

## Data Availability

The datasets analyzed during the current study are available from the corresponding author on reasonable request.

## Abbreviations

ACDF: Anterior cervical discectomy and fusion
ACDR: Artificial cervical disc replacement
ASD: Adjacent segment degeneration
CDDD: Cervical degenerative disc disease
COR: Center of rotation
CR: Center of reaction
FSU: Functional spinal unit
ICR: Instantaneous center of rotation
IROM: Inferior range of motion
mJOA: Modified Japanese Orthopedic Association
NDI: Neck Disability Index
ROM: Range of motion
SROM: Superior range of motion
VAS: Visual analogue scale
